# Supplementary figures and images for: Continuous speech with pauses inserted between words increases cortical tracking of speech envelope
Source: PLoS One. 2023 Jul 27;18(7):e0289288. doi: 10.1371/journal.pone.0289288 (PMC10374040; doi:10.1371/journal.pone.0289288)

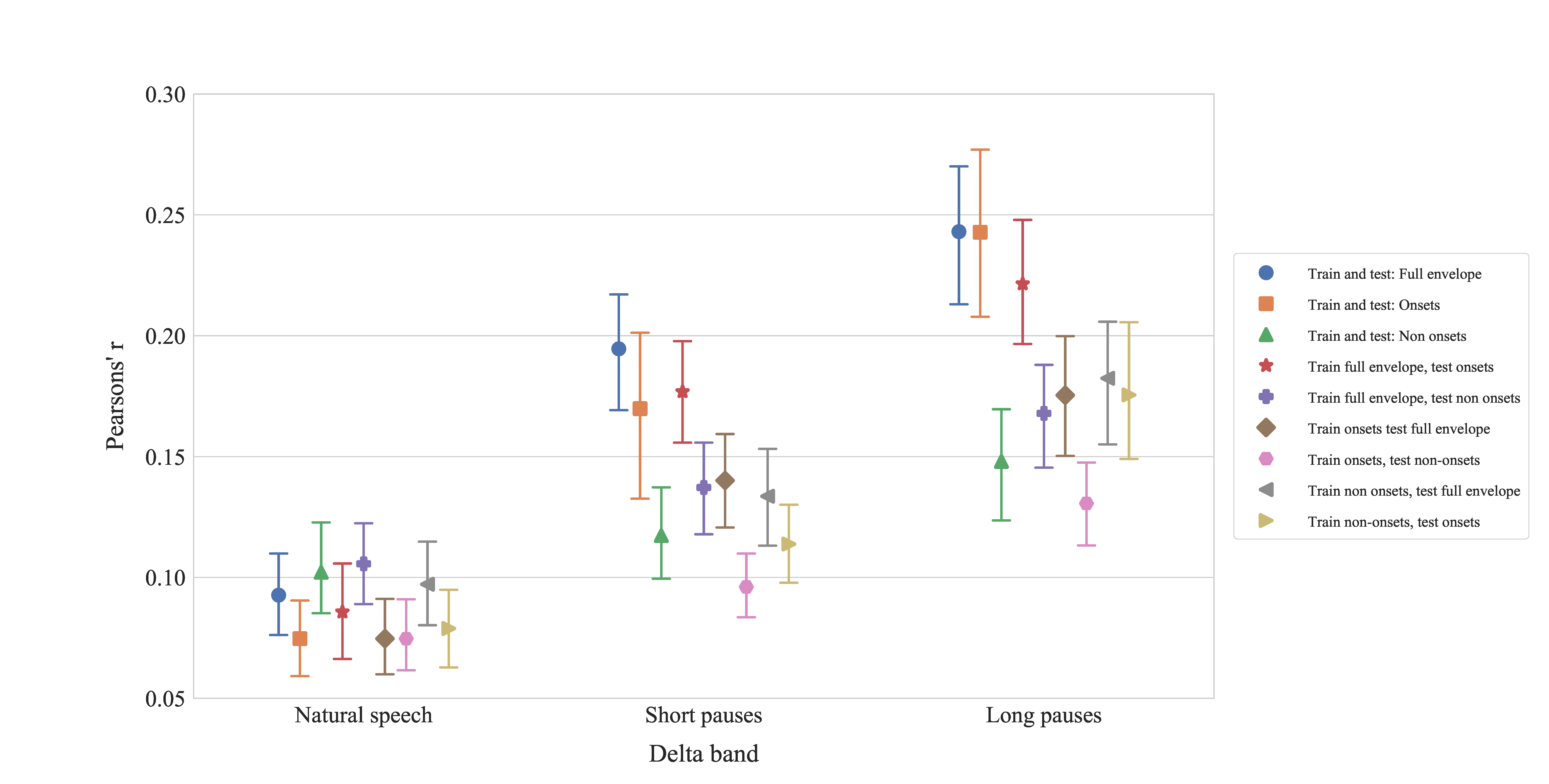

Supplement: S1 Fig — Each point indicates the average Pearsons’ r across sixteen participants. Error bars indicate the 95% confidence interval for the mean. (TIF) [file pone.0289288.s001.tif]

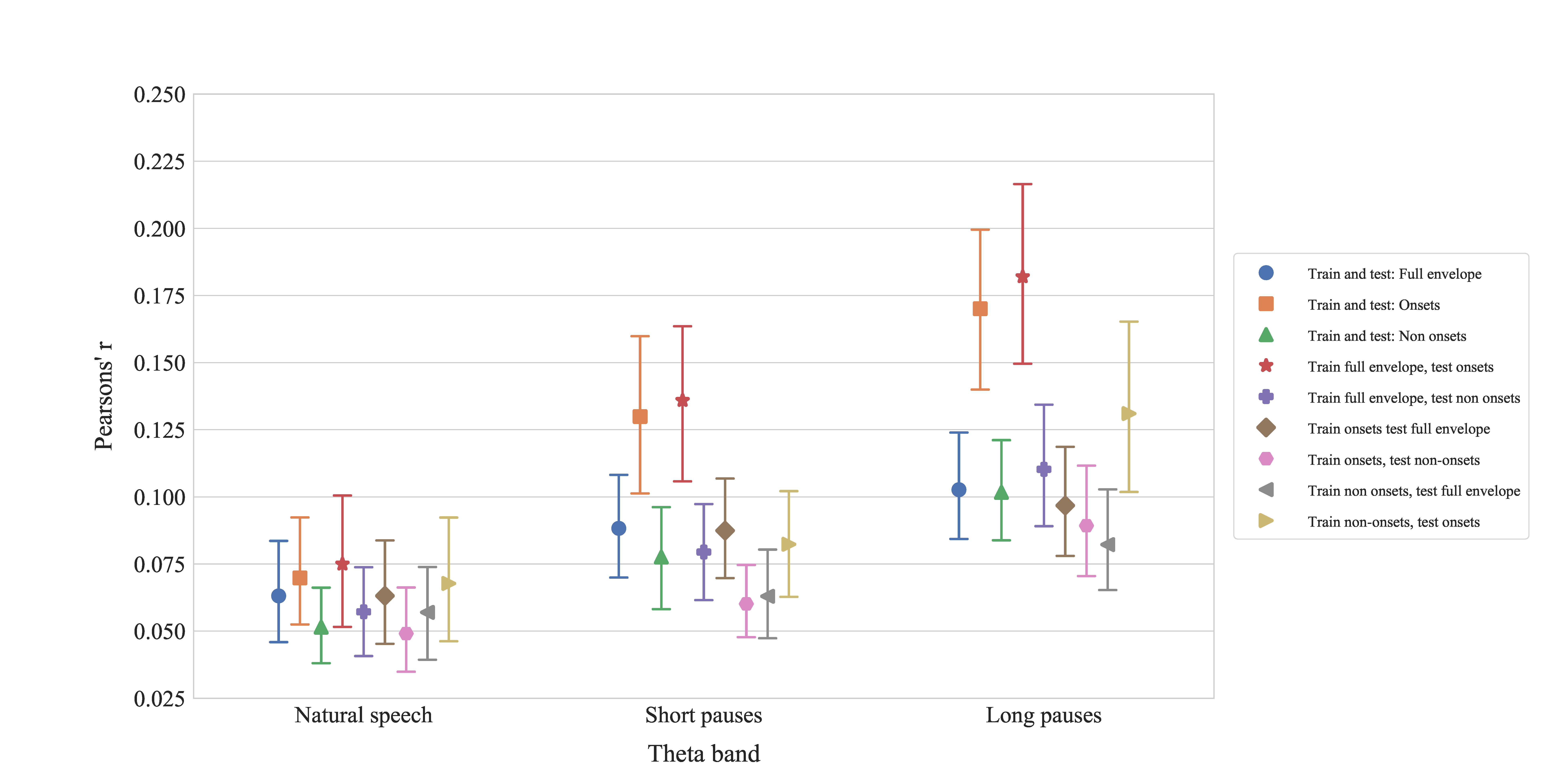

Supplement: S2 Fig — Each point indicates the average Pearsons’ r across sixteen participants. Error bars indicate the 95% confidence interval for the mean. (TIF) [file pone.0289288.s002.tif]
