# Supplementary material for: Continuous speech with pauses inserted between words increases cortical tracking of speech envelope
Source: PLoS One. 2023 Jul 27;18(7):e0289288. doi: 10.1371/journal.pone.0289288 (PMC10374040; doi:10.1371/journal.pone.0289288)
Supplement: Attachment — Submitted filename: Continuous Speech with Pauses - revised 2 - response.docx [file pone.0289288.s004.docx]

Manuscript number: PONE-D-22-18218R1

Response to reviewer’s comments

Dear Reviewers,

The authors thank you both reviewers for your consideration and valuable comments on the manuscript, title: “Continuous speech with pauses inserted between words increases cortical tracking of speech envelope”, submitting for publication in PLOS ONE. In this document, we have addressed the reviewer’s comments following the first revision in the following pages.

Please note that all the reviewer’s comments are in **bold text**.

Point-by-point responses to comments are indented normal text.

Changes in the manuscript are quoted in “*italic text” and indented*.

Line where changes were made are in [*red italics]*.

Reference number

26.	Chalas N, Daube C, Kluger DS, Abbasi O, Nitsch R, Gross J. Speech onsets and sustained speech contribute differentially to delta and theta speech tracking in auditory cortex. Cereb Cortex. 2023;33(10):6273-81. doi: 10.1093/cercor/bhac502.

was newly added to the reference list.

Best regards,

Suwijak Deoisres

**Reviewer #1:**

**The authors have addressed my comments from the previous manuscript. However, I still have a few further comments.**

**1 - Modulation spectrum of the stimuli (figure 2):
The 3 conditions show similar modulations around the theta-band range (4-8 Hz), but it is also evident that the 2 manipulated conditions show an additional peak in the delta-band range (< 4 Hz) mirroring their respective pause durations (lower frequency peak for long compared to short pauses). Although this is most probably an inevitable consequence of the fixed-duration pause manipulation, currently it remains unclear to what extent these delta-band acoustic modulations might impact the reported results. For example, could this explain why delta-band effects seem generally higher than theta-band effects? I am not necessarily asking for additional control analyses, but I think it is a fair point to be addressed in the manuscript.**

R1. We agree that the delta band modulation may strongly affect the cortical tracking in the delta band. We have carried out an additional control analysis to address this potential problem.

We added an additional section with the title *“Effect of delta band acoustic modulation on the cortical tracking of speech envelope” [lines 411-412]* in the results to report this control analysis, as follows:

(New figure) *“Fig 7 caption” [lines 413-416]*

*“As shown in Fig 2 that an additional peak in the modulation spectrum appears in the delta band frequency for speech with short and long pause inserted, it is unclear whether the relatively greater correlation coefficients in the delta band compared to the theta band was a result of the delta modulation rate or not. An additional analysis was conducted by removing samples in pause segments and samples in the EEG in lagged time series at the same sample index from the decoding process. This was done to only relate the EEG to the envelope where speech occurs, ensuring a consistent modulation spectrum across different speech pause conditions. This will be referred to as the pauses removed condition. We also included an additional pause removed decoding condition using longer EEG time lag, 0-500 ms, to examine if the original 0-300 ms time lag was sufficient to capture the effect of the delta band modulation rate.*

*Fig 7 shows the mean correlation coefficients averaged across all participants obtained from decoders trained and test on the full envelope and the envelope with pauses removed in three pauses conditions. Specifically for this analysis, the adjusted significance level for multiple comparisons was adjusted to p≤0.0056 for 9 pairwise comparisons (within each speech pause condition only) in each EEG frequency band. Overall, the same trend in increasing correlation coefficients when longer pauses were inserted to the speech stimulus as reported earlier remains but the correlation values changed significantly when pauses were removed from the decoding process. In the delta band, in the short and long pauses condition, the correlation coefficients when decoding with pauses removed were significantly lower compared to when decoding using the full envelope (p<0.001). While in the theta band, correlation coefficients from decoders with pauses removed were significantly greater than when decoding using the full envelope (p<0.001). Correlation coefficients from pauses removed decoders with different time lags, 0-300 ms and 0-500 ms, were not statistically significant in both the delta and theta band.*

*The greater correlation coefficients from the decoder in the delta band compared to the theta band appear to be partially affected by the delta band modulation rate in speech with inserted pauses. This is due to the significant decrease in correlation coefficients in the delta band when the pause segments are removed from the decoder analysis, as the EEG delta oscillation may persist in those segments. Despite this, the trend of increasing cortical tracking when pauses are inserted in speech remains. It is also evident that the effect of inserted pauses in speech is stronger for the cortical tracking of speech envelope in the delta band than in the theta band.” [lines 417-446]*

We also modified figure 2 by including the modulation spectrum of the stimuli when pauses were removed. [lines 188-192]

**2 - About the hypothesized effect of pauses (only based on Hamilton et al.’s findings), another recent article that could also support this hypothesis partially:
Chalas, Nikos, et al. "Speech onsets and sustained speech contribute differentially to delta and theta speech tracking in auditory cortex." Cerebral Cortex (2023).** [**https://doi.org/10.1093/cercor/bhac502**](https://doi.org/10.1093/cercor/bhac502)

R2. Thank you for sharing the article with us. We agree that the study by Chalas et al. partially supports the hypothesised effect of pauses in speech. We cited the recommended study, in addition to Hamilton et al.’s study. *[line 110]*

**3 - Lines 462-464: “However, it may be difficult to clearly quantify whether the cortical tracking of speech envelope when pauses were inserted to speech is dominated by the encoding of acoustic information or not.” I think this affirmation could be rephrased as future work rather than as a problem difficult to solve, considering the possibilities offered by the mTRF approach. In particular, encoding models with different sets of acoustic and/or linguistic regressors are commonly used in the literature to dissociate lower-level (e.g., acoustic) from higher-level (e.g., lexical or semantic components related to comprehension) contributions.**

R3. We agree that this should be a possible area for future studies to explore. We have revised the sentences as follows:

*“Future studies may consider implementing the encoding models to dissociate the contribution of lower-level (e.g., acoustic envelope) and higher-level (e.g., phonemes and phonetic features) information of speech to the cortical responses to speech with pauses [5].” [lines 502-504]*

**4 - Lines 561-562: “Cortical responses to natural speech in the delta band appear more strongly linked to non-onset segments, while theta band responses are more related to onset segments.”. This contradicts the lack of significant differences in the natural speech conditions reported in Figure 4.**

R4. We edited the sentence to avoid reporting contradictory results. The edited sentences are as follows:

*“Cortical responses to natural speech in the delta and theta band are not strongly related to either onset or non-onset segments. However, when pauses were introduced into the speech, responses in both frequency bands become dominated by the onsets.” [lines 601-603]*

**5 - Lines 564-567: “The results could be exploited in the clinic in detecting cortical tracking of speech envelope or to onset segments in speech more effectively, where the sensitivity to acoustic onsets could be an indication of how well a person can segment phrases and sentences during speech comprehension.”. I think this somehow contradicts the previous discussion about the uncertainty on the reported findings actually reflecting comprehension-related processes (like syntactic phrasing in this case). Also taking into account the reduced speech naturalness due to the implemented manipulation, what are the clinical implications of this study?**

R5. We agree that these sentences do not align with the previous discussion. It is also not completely clear how the modification of inserting pauses in speech should be exploited in the clinic, given the current findings. Instead, we have therefore changed the sentences to remind the reader that we are uncertain about how the effect of pauses links to speech comprehension, as follows:

*“The influence of the onset responses also led to increased number of cases where cortical tracking of speech envelope was significant, but this may not be an indication of better speech comprehension.” [lines 604-606]*

**6 - Minor comment: Figure numbers are missing in the manuscript. Also, figures 3 and 4 show the same results (with and without significance asterisks, respectively).**

R6. We apologise for the incorrect figure that was previously uploaded. The correct figure has been uploaded in its place.
